# Phytochemical Contents and Pharmacological Potential of *Parkia speciosa* Hassk. for Diabetic Vasculopathy: A Review

**DOI:** 10.3390/antiox11020431

**Published:** 2022-02-21

**Authors:** Ahmad Khusairi Azemi, Muhammad Luqman Nordin, Kamarul Ariffin Hambali, Nur Amalina Noralidin, Siti Safiah Mokhtar, Aida Hanum Ghulam Rasool

**Affiliations:** 1 Department of Pharmacology, School of Medical Sciences, Universiti Sains Malaysia (Health Campus), Kota Bharu 16150, Kelantan, Malaysia; akhusairi@usm.my (A.K.A.); safiahm@usm.my (S.S.M.); 2 Department of Veterinary Clinical Studies, Faculty of Veterinary Medicine, Universiti Malaysia Kelantan, Pengkalan Chepa, Kota Bharu 16100, Kelantan, Malaysia; luqman.n@umk.edu.my (M.L.N.); d20d015f@siswa.umk.edu.my (N.A.N.); 3 Faculty of Earth Science, Universiti Malaysia Kelantan, Jeli 17600, Kelantan, Malaysia; kamarul@umk.edu.my

**Keywords:** diabetes, hypoglycemic, antioxidant, anti-inflammatory, endothelial dysfunction

## Abstract

Diabetes mellitus (DM) is a metabolic disorder characterized by hyperglycemia and is considered a major health problem in the world. It is associated with endothelial dysfunction which causes progressive vascular damage. DM is a known risk factor for atherosclerosis and cardiovascular complications such as peripheral artery disease, coronary artery disease, and stroke. Medicinal plants may act as an alternative resource or adjunctive treatment option in the treatment of diabetes and its cardiovascular complications. *Parkia speciosa* (Fabaceae) is a plant found abundantly in the Southeast Asian region. Its seeds, with or without pods, and roots have long been used as a traditional medicine in this region to treat hypertension and diabetes. Studies have shown its numerous beneficial pharmacological properties. Extracts of *P. speciosa*, particularly from its seeds and empty pods, show the presence of polyphenols. They also exhibit potent antioxidant, hypoglycemic, anti-inflammatory, and antihypertensive properties. Its hypoglycemic properties are reported to be associated with the presence of β-sitosterol, stigmasterol, and stigmat-4-en-3-one. The current review aimed to provide an overview of the current status of *P. speciosa*, its pharmacological potential, and its phytochemical content in attenuating diabetic vasculopathy. Glycemic status, oxidative stress, inflammation, and hyperlipidemia are known to play pivotal roles in the initiation and severity of diabetic cardiovascular diseases; thus, targeting these factors might be beneficial for preventing and/or treating diabetic vasculopathy.

## 1. Introduction

Diabetes mellitus is a chronic and complex metabolic disorder of multiple etiologies with profound consequences, both acute and chronic [1,2]. Diabetes mellitus and its complications affect people both in developing and developed countries, leading to major health and socioeconomic challenges [2]. Diabetes can cause microvascular and macrovascular complications. Microvascular complications include retinopathy, neuropathy, and nephropathy. Macrovascular complications comprise coronary artery disease, cerebrovascular disease, and peripheral arterial disease [2,3].

*Parkia speciosa* Hassk., commonly called stinky bean, is an edible legume belonging to the Fabaceae family. It is commonly found in Malaysia, Thailand, Indonesia, Indonesia, Singapore, Borneo, Philippines, Madagascar, and Africa [4]. *P. speciosa* is commonly known as “petai” in Southeast Asian countries like Malaysia and Indonesia, and as “sator” or “sataw” in Thailand. In Malaysia, the seeds of *P. speciosa* are mostly eaten as “ulam”, which consists of raw or boiled seeds consumed with chilies, with prawn paste as the main ingredient. The mature seeds of *P. speciosa* are usually pickled in brine and are a popular pickle product in Malaysia [5]. Traditionally, these plant seeds have been used by locals to treat various diseases and symptoms like diabetes, kidney disorder, headache, hypertension, skin problems, and diarrhea [6,7,8]. Scientifically, *P. speciosa* has been reported to possess hypoglycemic [9,10,11], antioxidant [8,12,13,14,15], anti-inflammatory [16,17], and antihypertensive [18,19] properties. The polyphenols found in this plant include gallic acid, ellagic acid, catechin, quercetin, vanillic acid, and epicatechin [19].

In this review, we highlight the pharmacological effects of *P. speciosa*, which include hypoglycemic, hypolipidemic, antioxidant, anti-inflammatory, and antihypertensive effects. This information might be of benefit for future investigations on *P. speciosa*, to clarify the links between its pharmacological properties, phytochemical components, and medicinal uses and contribute to attenuating diabetic vasculopathy and its cardiovascular complications. 

## 2. Methods

A nonsystematic search of academic databases (PubMed, Web of Science, ScienceDirect, and Google Scholar) and grey literature (Google) was performed to extract and synthesize relevant studies that describe the potential roles of *P. speciosa* in the management of diabetes and related cardiovascular complications. The search terms used included “*P. speciosa*”, “*P. speciosa* AND antioxidant”, “*P. speciosa* AND diabetes”, “*P. speciosa* AND anti-inflammation”, “*P. speciosa* AND phytochemistry”, “diabetes”, “cardiovascular disease”, “inflammation”, and “diabetic vascular disease”. A total of 86 articles published from 1991 to 2021 were obtained. Articles that contained relevant and useful information regarding *P. speciosa* were included in this review. Only articles published in English were included. Information regarding traditional uses of and phytochemical compounds identified from *P. speciosa* was summarized in the form of two tables. 

## 3. Parkia speciosa

### 3.1. Taxonomical Classification

Kingdom: Plantae; Phylum: Trcheophyta; Class: Magnoliopsida; Order: Fabales; Family: Fabaceae; Genus: *Parkia*; Species: *P. speciosa*.

### 3.2. Botanical Description

**Figure 1:**
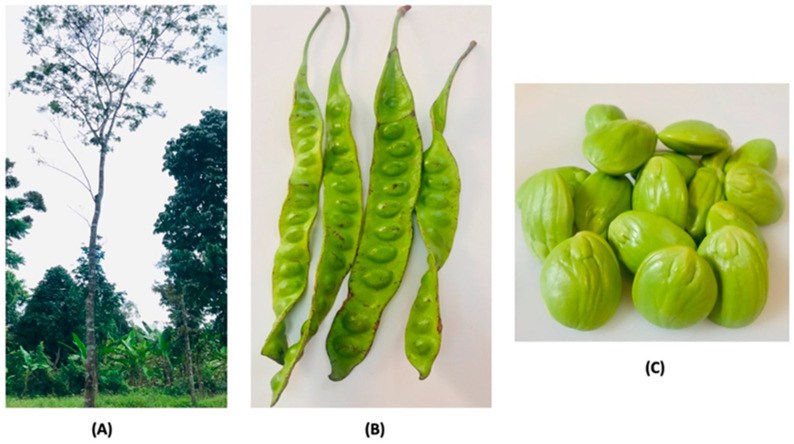
The tree (**A**), pods (**B**), and seeds (**C**) of *P. speciosa* plants.

*P. speciosa* thrives on podzolic sandy loam and in areas near riverbanks in primary lowland rainforests in Southeast Asia. The optimum annual temperature for the proliferation of the plant is 24 °C. The tree is cultivated in the plains up to an elevation of 1500 m. The plant is propagated via seed sowing, stem cutting, and budding [20,21]. The agronomic practices used for the pretreatment of seeds to overcome dormancy and accelerate seed germination are seed coat shelling or soaking of the seeds in water, ample light, and space provision [21]. Seeds are cut opposite to the micropyle to prevent damage to the embryo during seed coat cutting. One year after sowing, at a height of 0.5–1.0 m tall, the trees are transplanted to the field with a distance of 10 m between rows and 10 m between the plants (10 m × 10 m) [21]. The mature plant can grow up to 40 m in height and 1 m in stem circumference (Figure 1a). Its leaves are bipinnate and alternate. It is an ornamental, perennial, and fruit-bearing tree which starts to flower and produce fruit at the age of seven years. It usually flowers in January to March and inAugust to October each year. The flowers are bulb-shaped and droop at the stalk ends, while the fruits are green, flat, and oblong pods of 35–55 cm in length and 3–5 cm in width, beetling in bunches of 6–10 (Figure 1b,c). The seeds have a foul, peculiar, unique, and distinctive smell with an elliptical shape, and can be eaten raw, roasted, cooked, or blanched [5,8]. The seed has a unique taste similar to that of garlic; it has no burning spiciness or pungency, and also has a unique Shiitake-mushroom-like flavor [18]. *P. speciosa* has been reported to have bioactive compounds, particularly thiazoline-4-carboxylic acid, also known as thioroline, which is an amino acid that gives *P. speciosa* seed its sulfur smell [22,23].

### 3.3. Traditional Medicinal Uses of P. speciosa

*P. speciosa* is a valuable herbal plant consumed by the Southeast Asian communities as a cooking ingredient and for various medicinal purposes. In Malaysia, its’ seeds have always been a popular ingredient in cooking, where they are usually served with chili paste or “sambal”, dried shrimp, and chili pepper as a popular local delicacy. It can be cooked in chili paste mixed with seafood, boiled in coconut milk with a variety of vegetables, or added as an ingredient to many other dishes, including fried rice or stir-fried food [5,21]. It is traditionally used in several ways for the treatment of diabetes and hypertension [24,25,26,27]. It was reported previously that the *P. speciosa* seeds, pods, and roots were traditionally applied by indigenous communities in Peninsular Malaysia to treat diabetes and hypertension [27]. Furthermore, *P. speciosa* has also been consumed for the treatment of skin-related diseases such as eczema, skin ulcers, measles, leprosy, wound, dermatitis, chickenpox, scabies, and ringworm [8,28,29]. Table 1 summarizes the traditional uses of *P. speciosa*.

### 3.4. Phytochemistry

The bioactive compounds in plants can be classified into primary and secondary metabolites. Primary metabolites play a significant role in growth, development, or reproduction via molecules like amino acids, carbohydrates, and lipids [35,36]. The secondary metabolites derived from plants include flavonoids, alkaloids, saponins, triterpenes, tannins, and phytosterols; all of these are used by plants in their defense mechanisms [35,37]. *P. speciosa* also contains various types of polyphenols and flavonoids, which are believed to be potential sources of bioactive compounds beneficial to health. Table 2 documents the phytochemical compounds identified from different parts of the *P. speciosa* plant using different types of extraction solvents.

## 4. Pharmacological Potential of *P. speciosa* Extract in Attenuating Diabetic Vascular Complications

### 4.1. Antioxidant Properties

Oxidative stress describes the condition wherein an excessive production of reactive oxygen species (ROS) overwhelms endogenous antioxidant defense mechanisms. The elevation of ROS levels has a detrimental effect on cellular functions resulting from ROS-induced damage to enzymes, lipid membrane, and nucleic acids [43]. Several studies have reported a decreased level of superoxide dismutase (SOD) and glutathione peroxidase (GPx) in both clinical and experimental diabetes [44], indicating an impaired defense system for free radical scavenging. In diabetes, excess ROS are produced via several mechanisms include autoxidation of glucose, advanced glycation end product (AGE) formation, and the binding of AGEs to the AGE receptors [44].

In diabetes, hyperglycemia induces the production of ROS, particularly the superoxide anion in the endothelium of the blood vessels [45]. This causes overproduction of ROS, leading to increased oxidative stress, which increases the tendency of ROS to react with other molecules. Superoxide anion tends to scavenge NO in the endothelium to form peroxynitrite. Peroxynitrite reduces the activity and bioavailability of NO, leading to impairment of NO-mediated relaxation and subsequently endothelial dysfunction. The endothelium is a monolayer of endothelial cells that lines the interior surface of blood vessels and cardiac valves in the entire vascular system. It plays a significant role in vascular growth and remodeling, regulation of blood vessel integrity, metabolism, cell adhesion, angiogenesis, vascular permeability, tissue growth, homeostasis, and immune response [43,46]. Damage to the endothelium leads to increased endothelial cell permeability to low-density lipoprotein (LDL-C), which increases its uptake into the subendothelial space. This LDL-C undergoes oxidation, forming oxLDL-C; oxLDL-C is taken up by macrophages via scavenger receptors in phagocytosis and pinocytosis. The oxLDL-C then undergo esterification, leading to the formation of foam cells. Foam cells undergo an inflammatory process, causing migration and proliferation of smooth muscle cells into the intima, and then atherosclerotic plaque is formed. 

Studies by Baynes (1991) and Ramesh et al. (2012) have shown that diabetes-induced lipid peroxidation causes secondary chronic diabetes complications including atherosclerosis [47,48]. Oxidation of lipids in plasma lipoprotein and cellular membranes is associated with the development of vascular diseases in diabetes. Hyperglycemia in diabetes leads to overproduction of ROS, particularly superoxide anion. The excess amount of these free radicals leads to the oxidation of phospholipids (lipid peroxidation) at the cell membranes, resulting in the production of toxic byproducts such as aldehyde, particularly malondialdehyde (MDA) [49]. All of these processes contribute further to the development of fibrous plaque, the advanced lesions of atherosclerosis. Furthermore, oxLDL-C promotes eNOS protein binding with scavenger receptor CD36, which reduces the activity of eNOS and NO bioavailability in endothelial cells [50]. This situation leads to impairment of NO-mediated relaxation, thus resulting in endothelial dysfunction. Free radical species can be neutralized through dismutation or reduction by endogenous antioxidants like SOD and catalase, as well as through direct scavenging or electron transfer by exogenous antioxidants such as vitamin C and E [51,52].

#### 4.1.1. Evidence from In Vitro Studies

An in vitro study by Aisha et al. (2012) showed high 2,2-diphenyl-1-picrylhydrazyl (DPPH) radical scavenging activities in methanol extract of *P. speciosa* empty pods. From the same study, the total phenolic content in *P. speciosa* extract was 25.55 ± 1.57 GAE/100 mg [53]. Another study by Siow and Gan (2013) demonstrated that an extract of *P. speciosa* seed exhibited high DPPH radical scavenging and ferric-reducing antioxidant power (FRAP) activity [18]. An in vitro study by Ko et al. (2014) demonstrated the antioxidant properties of ethanolic and aqueous extracts of *P. speciosa* empty pods on antilipid peroxidation, DPPH radical scavenging activity, 2,2’-azino-bis-3-ethylbenzothiazoline-6-sulfonic acid (ABTS) radical scavenging, superoxide radical scavenging, metal chelating, and reducing power assays. In the study, ethanol extract showed more potent activities compared to aqueous extract in anti-lipid peroxidation, DPPH radical scavenging activity, ABTS radical scavenging, metal chelating activity, and reducing power activity [38]. 

Wonghirundecha et al. (2014) also found that *P. speciosa* pod extract showed high DPPH, ABTS radical scavenging, and metal ion chelating activity [54]. Sonia et al. (2018) reported that the hydromethanolic extract of *P. speciosa* pods showed high total phenolic, total flavonoids, and FRAP assay activity [55]. These data were supported by high DPPH radical scavenging activity and high hydrogen peroxide (H_2_O_2_) inhibition [55]. Another report by Ghasemzadeh et al. (2018) showed that the extract of *P. speciosa* seed produced high DPPH radical scavenging (66.29%) and FRAP activity [14]. 

#### 4.1.2. Evidence from In Vivo Studies

A study by al Batran et al. (2013) showed that administration of an ethanolic extract of *P. speciosa* leaf decreased the levels of MDA and increased SOD and total glutathione (GSH) levels in gastric tissue homogenate of a gastric ulcer rat model [56].

A recent study by Gao et al. (2021) reported the effects of *P. speciosa* empty pod ethanolic extract on antioxidant enzyme activities in the liver and kidney of diabetic rats. In that study, administration of *P. speciosa* extract at the dose of 100 mg/kg increased SOD (16.56 ± 2.03 vs. 9.19 ± 1.40 U/mg protein), glutathione peroxidase (GSH-Px; 106.76 ± 2.20 vs. 78.29 ± 14.48 U/mg protein), and catalase (CAT; 52.98 ± 4.95 vs. 40.59 ± 6.08 U/mg protein) and reduced MDA levels (20.37 ± 3.25 vs. 36.42 ± 8.01 nmol/mg protein) in liver tissues of treated diabetic rats compared to diabetic controls. In the same study [57], treatment with a higher dose of extract (400 mg/kg) was associated with increased SOD (18.10 ± 1.52 vs. 9.19 ± 1.40 U/mg protein) and GSH-Px (206.19 ± 19.29 vs. 78.29 ± 14.48 U/mg protein), CAT (68.20 ± 3.97 vs. 40.59 ± 6.08 U/mg protein) and reduced MDA (17.65 ± 2.38 vs. 36.42 ± 8.01 nmol/mg protein) in the liver tissues of diabetic rats compared to diabetic controls. In addition, the effects of *P. speciosa* extract on antioxidant enzyme levels were significantly improved in the kidney tissues of treated diabetic rats compared to diabetic controls [57].

Thus, in vitro studies have shown antioxidant properties of various *P. speciosa* extracts as assessed via DPPH, FRAP, and ABTS radical scavenging tests. This is supported by high antioxidant enzyme levels reported by in vivo studies in liver and kidney tissue homogenates. The antioxidant properties of *P. speciosa* may provide a beneficial effect on cardiovascular complications of diabetes, for example by improving NO bioavailability. Currently, there are no studies on the effects of *P. speciosa* extracts on vascular tissue oxidative stress. 

### 4.2. Hypoglycemic Properties

Diabetes is associated with endothelial dysfunction. Endothelial dysfunction is an early step in the development of atherosclerosis. It is characterized by impaired endothelium-dependent relaxation, which occurs due to the imbalance between vasodilation and vasoconstriction due to a reduction in the bioavailability of the vasodilator NO [58]. In diabetes, hyperglycemia increases ROS generation via glucose autoxidation and AGEs. Increased ROS production augmented its tendency to react with NO to form cytotoxic oxidant peroxynitrite. Peroxynitrite leads to degradation of eNOS cofactor tetrahydrobiopterin (BH4), causing the uncoupling of eNOS, thus reducing NO production [59]. Reduced NO bioavailability also upregulates adhesion molecules such as intracellular adhesion molecule (ICAM), vascular adhesion molecule (VCAM), and E-selectin in endothelial cells via induction of nuclear factor kappa B (NF-kB) expression [60]. Increase in adhesion molecules initiate the inflammatory process that leads to atherosclerotic plaque formation. Furthermore, protein kinase C (PKC) activation due to hyperglycemia may affect eNOS activity in endothelial cells. PKC activates nicotinamide adenine dinucleotide phosphate (NADPH) oxidase to generate more free radical species. These free radical species reduce the antioxidant effect of SOD especially the scavenging of superoxide anion, thus increasing the production of peroxynitrite. These were confirmed by studies that showed vascular SOD activities in the aorta of diabetic rats were reduced compared to non-diabetic rats, indicating an increase in oxidative stress [58,61].

#### 4.2.1. Evidence from In Vitro Studies

The hypoglycemic property of *P. speciosa* was also tested in vitro by measuring the activities of α-glucosidase and α-amylase enzymes. α-glucosidase is an enzyme that increases postprandial glucose levels by hydrolyzing carbohydrates into glucose in the small intestine [51,62]. α-amylase is an enzyme that breaks down carbohydrates into simpler saccharides [7]. Inhibition of the α-glucosidase and α-amylase enzymes in the small intestine is an effective approach for the management of diabetes [51]. Therefore, a natural product that inhibits α-glucosidase and α-amylase becomes targets in the search for new compounds in the management of diabetes [51,63].

Tunsaringkarn et al. (2008) has reported α-glucosidase inhibitory activity of Thai mimosaceous plant extracts which includes *P. speciosa* [64]. In that study, the petroleum ether, dichloromethane, and ethanol extracts of *P. speciosa* seed showed 10.24%, 10.57%, and 45.72% α-glucosidase inhibition respectively. In addition, in the same study, the petroleum ether, dichloromethane, and ethanol extracts of *P. speciosa* pericarp showed 18.62%, 21.93%, and 89.46% α-glucosidase inhibition respectively [64]. In 2009, Tunsaringkarn and colleagues have demonstrated the α-glucosidase inhibitory activity of the aqueous extract of *P. speciosa* seeds and pericarp [65]. From this study, aqueous extract of *P. speciosa* seed showed 4% α-glucosidase inhibition, while aqueous extract of *P. speciosa* pericarp showed 61.86% inhibition at the same dose (1 mg/mL) [65]. 

Sonia et al. (2018) reported an in vitro study on the anti-diabetic activity of *P. speciosa* seeds extract via α-amylase and pancreatic lipase inhibitory actions [55]. In that study, *P. speciosa* extract showed maximum α-amylase inhibition of 79.2% at 500 μg/mL. The IC_50_ of α-amylase inhibition of *P. speciosa* extract and the hypoglycemic drug acarbose were found to be 199.29 μg/mL and 324.18 μg/mL. Other than that, *P. speciosa* extract showed high pancreatic lipase inhibition of 89.5% at 500 μg/mL. The IC_50_ of pancreatic lipase inhibition of *P. speciosa* extract and standard dose acarbose was found to be 196.61 μg/mL and 227.27 μg/mL [55]. Therefore, by having α-glucosidase and α-amylase inhibitory properties, *P. speciosa* extracts have the potential to be developed as a hypoglycemic agent. 

#### 4.2.2. Evidence from In Vivo Studies

In alloxan-induced diabetic rats, chloroform extract of *P. speciosa* seeds and empty pods significantly reduced glucose levels 2 h after ingestion, and the effect lasted for at least 24 h [11]. In that study, normal rats had an average blood glucose concentration of 124 mg/100 mL, while the diabetic rats average blood glucose concentration was 379 mg/100 mL after ingesting 1 g glucose/kg of body weight. Oral administration of 0.4 g/kg of *P. speciosa* ground pods reduced 36% of blood glucose concentration in diabetic rats. In addition, oral administration of 0.4 g/kg of *P. speciosa* ground seeds reduced 57% of blood glucose concentration in diabetic rats [11]. 

Jamaluddin et al. (1994) demonstrated the hypoglycemic property of bioactive compounds isolated from chloroform extract of *P. speciosa* seeds [9]. In that study, a tested fraction isolated from the crude extract of *P. speciosa* seeds which contained a mixture of *β*-sitosterol (66%) and stigmasterol (34%) showed 83% hypoglycemic activity at the dose of 100 mg/kg (173 mg glucose/100 mL blood) compared to untreated diabetic rats (400 mg glucose/100 mL blood). In the same study, treatment with glibenclamide (positive control) at a dose 5 mg/kg reduced blood glucose concentration by 111% (98 mg glucose/100 mL blood), which was below the blood glucose level in non-diabetic rats (125 mg glucose/100 mL blood) [9].

In another study, Jamaluddin and collogues showed the hypoglycemic property of stigmast-4-en-3-one isolated from chloroform extract of *P. speciosa* empty pods, and from that study, administration of stigmast-4-en-3-one reduced 84% blood glucose concentration (170 mg glucose/100 mL blood). In the same study, administration of 5 mg/kg of glibenclamide in diabetic rats lowered the blood glucose concentration by 111% (to 98 mg glucose/100 mL blood), to below the glucose levels of non-diabetic rats [66].

A recent study by Gao et al. (2021) demonstrated hypoglycemic property of ethanol extract of *P. speciosa* empty pods in high-fat diet-induced type-2 diabetic rats. Treatment at low (100 mg/kg) and high doses (400 mg/kg) significantly reduced fasting blood glucose concentrations to 12.1 mmol/L and 10.82 mmol/L compared to diabetic controls (29.07 mmol/L) respectively. The effect of *P. speciosa* extract in reducing blood glucose levels was comparable with the anti-diabetic drug glibenclamide at a dose of 10 mg/kg (12.40 mmol/L) [57].

### 4.3. Hypolipidemic Properties

Hyperlipidemia increases cardiovascular risk in diabetes. It is associated with alterations in the physical properties of cellular membranes, which facilitate the escape of free radicals from the electron transport chain or activation of NADPH oxidase [67,68]. These cause an augmented free radicals species generation and increase their tendency to react with other molecules. This results in oxidation and peroxidation of lipids, protein, and lipoprotein [69]. Peroxidation of the membrane of endothelial cells lead to endothelial damage and dysfunction. In a hyperlipidemic state, the presence of caveolin-1 in the plasma membrane of endothelial cells contributes to endothelial dysfunction. Caveolin-1 is an integral membrane protein, which is the principal component of caveolae in membranes and is involved in multiple cellular functions such as endocytosis, cholesterol homeostasis, and signal transduction [70]. In the hyperlipidemic state, caveolin-1 binds to eNOS protein leading to eNOS deactivation [50]. This results in a reduction of NO bioavailability which promote endothelial dysfunction and atherosclerotic lesion formation. Oxidation of LDL-C (oxLDL-C) in the arterial wall is an crucial step in endothelial dysfunction and atherogenesis. In hyperlipidemic conditions, oxLDL-C causes an increase in the binding of eNOS protein to the scavenger receptor CD36, which attenuates the activity of eNOS and reduces eNOS protein expression at endothelial cells [50]. Therefore, reducing the level of LDL-C and inhibiting LDL-C oxidation are likely to be able to prevent the initiation of atherosclerosis.

#### Evidence from In Vivo Studies

A study by Tandi et al. (2020) showed the effects of ethanol extract of *P. speciosa* peels on total cholesterol levels in the hypercholesterolemia rat model [71]. In that study, oral administration at the dose of 300 mg/kg daily reduced 52.73% of total cholesterol levels in hypercholesterolemia mice compared to the negative control group after 35 days. The effect of *P. speciosa* extract was comparable with the effect of simvastatin, which reduced total cholesterol levels by 57.27% [71].

Gao et al. (2021) demonstrated that administration of ethanol extract of *P. speciosa* empty pods at doses of 100 mg/kg and 400 mg/kg improved the serum lipid profile in a type 2 diabetic rat model fed with a high-fat diet [57]. Administration of the extract at a low dose (100 mg/kg) significantly reduced total cholesterol, triglycerides, LDL-C, and increased high-density lipoprotein cholesterol compared to untreated diabetic control. In addition, in the same study, treatment at a higher dose (400 mg/kg) also reduced total cholesterol, triglycerides, LDL-C, and increased high-density lipoprotein cholesterol (HDL-C) compared to untreated diabetic controls. These effects of *P. speciosa* were comparable with the effects of glibenclamide (10 mg/kg) [57].

### 4.4. Anti-Inflammatory Properties

Inflammation is a contributor to endothelial dysfunction and plays a significant role in the development of atherosclerosis. It modifies the synthesis and degradation of vasodilators and vasoconstrictors. At the intima layer of the arterial cell wall, monocytes release several inflammatory cytokines during hyperglycemic state such as interleukins-1 (IL-1), IL-1β, IL-6 and tumor necrosis factor-alpha (TNF-α); all of which contribute to vascular endothelium injury and induce vascular wall plaque formation [72]. TNF-α activates NADPH oxidase resulting in increased superoxide anion production, leading to a reduction of NO production and bioavailability [73]. It also reduces the expression of eNOS via enhanced superoxide anion generation and affects the half-life of eNOS mRNA, thus further reducing NO bioavailability [74,75]. NO protects blood vessels from endogenous injury by inhibiting leukocyte and platelet interactions with the vascular wall and preventing vascular smooth muscle (VSMC) proliferation and migration [76,77]. A reduction in endothelium-derived NO increases the activity of nuclear factor kappa-light-chain-enhancer of activated B cells (NF-κB), resulting in over-expression of leukocyte adhesion molecules and an increase in the production of inflammatory cytokines particularly TNF-α. These activities promote VSMC and monocytes migration into the intima and the formation of foam cells, characterizing the initial morphological changes of atherosclerosis [78]. Studies had demonstrated an augmented level of the pro-inflammatory cytokine TNF-a in aortic and cardiac tissues of diabetic rat models [79,80]. Hyperglycemia leads to an increase in TNF-a levels due to increased free radicals. TNF-a activates NADPH oxidase that increases the generation of superoxide anion. Overproduction of superoxide anion increases its tendency to scavenge NO to form peroxynitrite [81]. Peroxynitrite disrupts the activity of the eNOS cofactor (BH4), leading to eNOS uncoupling, thus reducing the potent vasodilator NO production [82].

#### Evidence from In Vitro Studies

An in vitro study by Mustafa et al. (2018) demonstrated the anti-inflammatory property of *P. speciosa* empty pod extract in human umbilical vein endothelial cells (HUVECs) [17], administration of *P. speciosa* extracts significantly reduced ROS levels, NF-kB p65, cyclooxygenase-2 (COX-2), and VCAM-1 protein expressions [17]. The effects of *P. speciosa* were comparable with the effect of quercetin in reducing these inflammation markers. Gui et al. (2019) showed the anti-inflammatory effects of *P. speciosa* empty pod extract in cardiomyocytes (H9c2) exposed to TNF-α. administration of *P. speciosa* significantly increased H9c2 cell viability, reduced NF-kB p65, p38 mitogen-activated protein kinase (MAPK), COX-2, VCAM-1 protein expression, and ROS levels [16]. Reduced levels of these inflammation markers can prevent/attenuate atherosclerotic plaque formation and its complications. Currently, there are no in vivo studies on the anti-inflammatory properties and vascular tissue inflammation in animal models.

### 4.5. Antihypertensive Properties

Hypertension is the most common preventable risk factor for cardiovascular diseases, including stroke, heart failure, coronary artery disease, myocardial infarction, and peripheral artery disease [83]. The pathogenesis of hypertension is multifactorial. Activation of the renin–angiotensin–aldosterone system (RAAS) is one of the important factors. Renin converts angiotensinogen to angiotensin I, which is then further converted to angiotensin II (Ang II) by the angiotensin-converting enzyme (ACE). Ang II causes an increase in aldosterone secretion from the adrenals which leads to sodium and water retention, therefore increasing blood pressure [19]. Ang II is also a potent vasoconstrictor that acts on the angiotensin II receptor on vascular smooth muscles, causing vasoconstriction. Ang II also activates NADPH oxidase activity, a major source of superoxide anion. These free radicals increase oxidative stress and can reduce the bioavailability of NO, resulting in impaired vasodilatation and then hypertension [19,84].

Antihypertensive agents can be very beneficial in reducing the risk of atherosclerosis, an artery-clogging process that causes heart failure and strokes. ACE inhibitors are a group of drugs widely used in the treatment of hypertension and congestive heart failure. These drugs inhibit ACE, an important element of the RAAS [85].The mechanisms of action for this group of drugs include reducing vascular resistance as well as decreasing blood volume, which leads to lowered blood pressure and decreased oxygen demand from the heart. 

#### 4.5.1. Evidence from In Vitro Studies

A study by Siow and Gan (2013) showed the antihypertensive properties of bioactive peptides of *P. speciosa* seed. In this study, protein hydrolysates from *P. speciosa* seed were shown to have the ability to inhibit ACE activity in the range of 50.6% to 80.2% (after 5× dilution), whereas the nonhydrolyzed samples did not show any ACE-inhibitory activity [18]. Another in vitro study by Khalid and Babji (2018) also demonstrated the ACE inhibitory activity of *P. speciosa*. In that study [13], an aqueous extract of *P. speciosa* seed showed 33.89% ACE inhibitory activity. 

A current in vitro study by Siti et al. (2021) reported the effect of ethanol extract of *P. speciosa* empty pod on cardiomyocyte hypertrophy in H9c2 cells [86]. In the study, treatment with *P. speciosa* extracts prevented Ang II-induced increases in cell size, NADPH oxidase activity, B-type natriuretic peptide levels, and ROS and reduced SOD activity. *P. speciosa* extract also reduced the levels of phosphorylated extracellular-signal-related kinase, p38, and c-Jun N-terminal kinase. All the effects of *P. speciosa* extract in the study were comparable with the effects of valsartan (an antihypertensive drug). 

#### 4.5.2. Evidence from In Vivo Studies

A study by Kamisah et al. (2017) reported the antihypertensive effect of methanolic extract of *P. speciosa* empty pods in a hypertensive rat model induced by L-NAME [19]. In that study, oral administration of *P. speciosa* extract at the dose of 800 mg/kg reduced systolic blood pressure, ACE, NADPH oxidase activity, and cardiac lipid peroxidation (TBARS) levels in the hypertensive rat model. In addition, administration of *P. speciosa* extracts also increased plasma NO levels (12.3%) in the hypertensive rat model. The effects of *P. speciosa* extract were comparable with the effects of nicardipine (3 mg/kg) in the hypertensive rat. Therefore, evidence from in vitro and in vivo antihypertensive studies show its potential benefit as an antihypertensive agent; thus, the potential of *P. speciosa* in the treatment of diabetic vasculopathy, atherosclerosis, and cardiovascular complications should be further explored.

## 5. Conclusions

Based on in vitro and in vivo studies, *P. speciosa* extracts possess hypoglycemic, hypolipidemic, antioxidant, anti-inflammatory, and antihypertensive properties. These properties have the potential to ameliorate diabetes-induced complications, particularly vascular endothelial dysfunction and atherosclerosis. These pharmacological effects may be associated with the presence of bioactive compounds in *P. speciosa*, including β-sitosterol, stigmasterol, and stigmast-4-en-3-one. Since *P. speciosa* is generally consumed orally, further clinical trials should also be performed to establish the clinical safety and efficacy of these plants for use as a treatment or adjunct in the treatment of diabetes and other cardiovascular diseases. More studies are also required to standardize the appropriate dosage of *P. speciosa* bioactive compounds.

## Figures and Tables

**Table 1:** Traditional uses of *P. speciosa.* Adapted from Saleh et al. (2021) [8].

Plant Part	Method of Preparation	Traditional Uses	Region/Country	References
Seeds	Eaten raw or cooked oral decoction	Diabetes	Malaysia	[8,27]
	Eaten raw	Diabetes	Singapore	[8,30]
	–	Loss of appetite	Indonesia	[31]
	Cooked	Kidney disorder	West Malaysia	[32]
Leaves	Pounded with rice and applied on the neck	Cough	Malaysia	[33]
	Decoction	Dermatitis	Indonesia	[8,28]
	–	Dermatitis	Indonesia	[31]
Root	Decoction	Skin conditions	Southern Thailand	[29]
	Decoction is taken orally	Hypertension and diabetes	Malaysia	[27,33]
	Oral decoction	Toothache	Malaysia	[34]

**Table 2:** Phytochemical compounds extracted from *P. speciosa* plants. Adapted from Saleh et al. (2021) [8].

Polyphenols	Plant Part	Extract	Country	References
Quercetin, rutin, kaempherol, catechin, luteolin, myricetin, gallic acid, caffeic acid, ferulic acid, trans-cinnamic acid, *p*-coumaric acid	Seed	Ethanol	Malaysia	[14]
Gallic acid, catechin, chlorogenic acid, vanillic acid, caffeic acid, epicatechin, kaempferol, ellagic acid, cinnamic acid, ferulic aid, *p*-coumaric acid, quercetin	Pod	Aqueous, ethanol	Malaysia	[38]
Lupeol, *β*-sitosterol, stigmasterol, stigmasterol methyl ester, stigmasta-5,24(28)-diene-3-ol, campesterol, arachidonic acid, linoleic acid chloride, linoleic acid, squalene, lauric acid, stearic acid, oleic acid, myristic acid, lanthionine, ethyl linoleate, ethyl stearate, 3-ethyl-4-nonanol, eicosanoic acid, elaidic acid, 2-nonade-canone, 2-pyrrolidi-none, 2-decanal, cyclo-decanone-2,4-decadienal, Hexaminde, vitamin E	Seed	Supercritical carbon dioxide	Malaysia	[39]
Apigenin, nobiletin, tangeritin, rutin, didymin, punicalin, coutaric acid, caftaric acid, malvidin, primulin	Pod	Methanol	Malaysia	[19]
*β*-sitosterol, stigmasterol, stigmasterol methyl ester, campesterol, arachidonic acid, linoleic acid, linoleic acid chloride, linoleic acid, squalene, stearic acid, oleic acid, palmitic acid, myristic acid, undecanoic acid, stearolic acid, linoleaidic acid methyl ester	Seed	–	Malaysia	[40]
1,3-dithiabutane, 2,4-dithiapenthane, 2,3,5-trithiahexane, 2,4,6-trithiaheptane, pentanal	Seed	Aqueous	Indonesia	[41]
1,2,4-trithiolane, 1,3,5-trithaine, 3,5-dimethyl-1,2,4-trithiolane, dimethyl tetrasulfid, 1,2,5,6-tetrahio-cane, 1,2,3,5-tetrathiane, 1,2,4,5-tetrathiane, 1,2,4,6-tetrathie-pane, lethionine	Seed	Hexane	Singapore	[42]
